# Polyvinylidene Fluoride Aerogels with Tailorable Crystalline Phase Composition

**DOI:** 10.3390/gels8110727

**Published:** 2022-11-09

**Authors:** Jorge Torres-Rodriguez, Diana E. Bedolla, Francesco D’Amico, Ann-Kathrin Koopmann, Lisa Vaccari, Giulia Saccomano, Richard Kohns, Nicola Huesing

**Affiliations:** 1 Chemistry and Physics of Materials, Paris-Lodron-University of Salzburg, Jakob Haringer-Str. 2A, 5020 Salzburg, Austria; 2 Salzburg Center for Smart Materials, Jakob Haringer-Str. 2A, 5020 Salzburg, Austria; 3 Elettra-Sincrotrone Trieste, SS14 Km 163.5, 34149 Trieste, Italy; 4 Area Science Park, Padriciano 99, 34149 Trieste, Italy; 5 Department of Engineering and Architecture, University of Trieste, Via Alfonso Valerio 6/1, 34127 Trieste, Italy

**Keywords:** aerogel, PVDF, crystalline phases, polyvinylidene fluoride

## Abstract

In this work, polyvinylidene fluoride (PVDF) aerogels with a tailorable phase composition were prepared by following the crystallization-induced gelation principle. A series of PVDF wet gels (5 to 12 wt.%) were prepared from either PVDF–DMF solutions or a mixture of DMF and ethanol as non-solvent. The effects of the non-solvent concentration on the crystalline composition of the PVDF aerogels were thoroughly investigated. It was found that the nucleating role of ethanol can be adjusted to produce low-density PVDF aerogels, whereas the changes in composition by the addition of small amounts of water to the solution promote the stabilization of the valuable β and γ phases. These phases of the aerogels were monitored by FTIR and Raman spectroscopies. Furthermore, the crystallization process was followed by in-time and in situ ATR–FTIR spectroscopy. The obtained aerogels displayed specific surface areas > 150 m^2^ g^−1^, with variable particle morphologies that are dependent on the non-solvent composition, as observed by using SEM and Synchrotron Radiation Computed micro-Tomography (SR-μCT).

## 1. Introduction

Polyvinylidene fluoride (PVDF) is a chemically stable, inert, and piezoelectric polymer [1]. Derived from these properties, this semicrystalline fluoropolymer finds applications in widespread fields such as catalysis [2], biomedicine [3], sensing technologies [4], and many more. In the field of sensor technologies, PVDF is of remarkable interest since it presents the highest piezoelectric constant of a polymer, good flexibility, and low density, which are required features for wearable, wireless, and self-powered sensors [4,5,6]. The semi-crystalline phase may comprise five different polymorphs (α, β, γ, δ, and ε), with α, β, and γ crystalline phases being the most predominant ones [7,8,9,10]. The α phase is easy to obtain since it is kinetically stable during crystallization, but its non-polar character produces an electrically inactive compound, as the electric dipoles are neutralized due to alternating trans-gauche conformation (TGTG’) [7,8,9]. Intended piezoelectric PVDF-based sensors are expected to contain mainly β, and γ phases since they display highly polar molecular conformations. Particularly, the atoms in the β phase are all trans-conformation (TTTT), thus exhibiting the highest net dipole moment resulting in a high piezoelectric constant [7,10]. On the other hand, the γ phase is structured in a gauche conformation for every fourth repeating unit (T_3_GT_3_G´), showing the higher Curie temperature, breakdown strength, and discharge energy, making it the best performer for large-scale applications under severe working conditions [7,11]. Hence, careful phase control of the PVDF-based materials is required.

Several synthetic approaches are focused on obtaining PVDF materials with a high content of the polar phases, including melting, mechanical stretching [12,13,14], annealing [15,16], blending [17,18], or addition of fillers [19,20,21], among others. However, such processing and fabrication techniques are impractical for certain materials, or they are not compatible with the final device design, thus stressing the importance of the exploration of alternative routes such as a precise crystal growth control [16,17,22,23,24]. Currently, different methods are being investigated to prepare PVDF materials such as fibers, films, or membranes exhibiting good piezoelectric properties; nonetheless, their performance is still far from alternative dense inorganic piezoelectric counterparts. It has been proven that induced porosity on PVDF materials significantly improves the piezoelectric response of the system [3,25,26]. However, producing porous PVDF materials is an even bigger challenge since the current production methods result in non-controlled porosity, large cavities, and difficulties to remove the template, therefore compromising the piezoelectric potential [25,26]. It is even more challenging to simultaneously optimize the porosity and specific phase configuration in high-quality porous PVDF bulk materials.

In this work, we tackled these challenges by developing a direct method to produce highly porous bulk PVDF aerogels with precise control of the phase composition, without further sample processing or the incorporation of a template. The limited available research on PVDF aerogels is focused on porous membranes [27,28,29], adsorbent media [30,31,32], composites [33,34,35], thermal insulation [36], and airborne particle filtration [16,37], mostly prepared by phase separation approaches using different solvents, followed by supercritical drying process, or in some cases by freeze drying, without meaningful attention on controlling the crystalline phases, somehow demonstrating that this porous system is seemingly ignored for piezoelectric applications. Consequently, an in-depth study on phase control has not been reported yet.

Our results indicate that a precise control of choice and amount of the components in the precursor solution allows for an excellent control of the crystalline phases, surface morphology, and physicochemical properties in highly porous aerogel monoliths. Namely, the interplay between the polymer, the solvent, and the gelation-induced crystallization must be optimized to produce high-quality PVDF aerogels. This contribution provides an easy and effective approach to crossover the outstanding physicochemical properties of aerogels with the efficient electroactive response of the PVDF, resulting in PVDF aerogels with adjustable characteristics that might be extrapolated to other porous PVDF-based systems.

## 2. Results and Discussion

In the first set of experiments, the gel formation of PVDF was examined in pure DMF solutions. The main focus was set on the concentration of PVDF that is necessary to form stable three-dimensional gel bodies and on the investigation of the resulting phase composition, as well as structural parameters such as specific surface area, porosity, and particle sizes.

*Gel formation*. To determine the gelation boundaries, amounts of PVDF (3, 5, 7, 9, and 12 wt.%) were dissolved in DMF at 80 °C. Subsequently, the solutions were transferred into sealed plastic containers to cool down and monitor the gel point. As presented in Table 1, gelation is not observed for samples with lower amounts than 9 wt.% of PVDF. Instead, liquid–liquid phase separation occurs with limited particle/chain connection [38], and therefore, a three-dimensional network is not formed. On the contrary, the 9 and 12 wt.% solutions turned into a rigid whitish wet gel. In this case, the system undergoes a spinodal decomposition in a colloid-rich region [9,39]. Thus, the 9, and 12 wt.% gels (hereafter labeled as P9 and P12, respectively) were submitted to a supercritical drying process to obtain PVDF aerogels.

**Figure 1:**
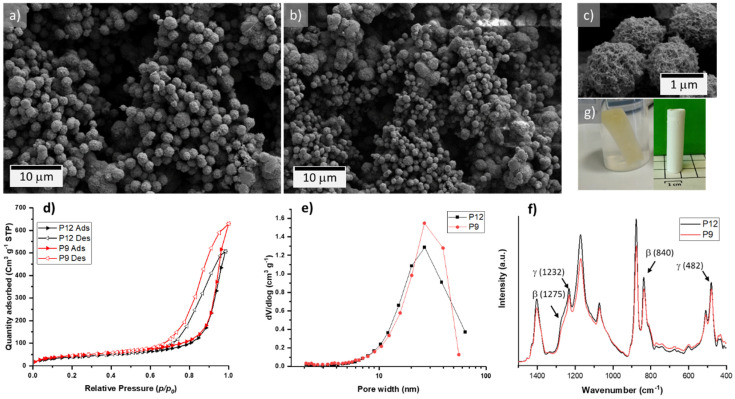
(**a**,**b**) SEM micrographs of the P12 (**a**) and P9 (**b**) aerogels, respectively; (**c**) spherulitic particles (P12) of the PVDF aerogels; (**d**) nitrogen sorption isotherms; (**e**) BJH pore size distribution plots; (**f**) FTIR spectra; and (**g**) obtained P12 wet gel and its aerogel counterpart upon supercritical drying.

After solvent extraction, an interconnected three-dimensional network of spherical particles of ~1–2 μm, as determined by SEM imaging, builds the P12 and P9 aerogels (Figure 1a–c) with no apparent differences between the two samples. The recorded N_2_ adsorption–desorption curves display type IV isotherms with H1-type hysteresis loops, which are characteristic of mesoporous materials [40] (Figure 1d). The distinctive spherulitic microstructure of both aerogels develops an S_BET_ of 145 and 168 m^2^ g^−1^ for P12 and P9, respectively (Table 1). The higher surface area of the 9 wt.% aerogel is attributed to the lower PVDF concentration in comparison to the P12 sample. This concentration difference slightly varied the density and the porosity of both aerogels, as observed in Table 1, but there is no noticeable difference in the pore size distribution, which shows a maximum at 23 nm for both mesoporous samples (Figure 1e).

FTIR measurements were carried out to determine the present crystalline phases; the recorded patterns for the P12 and P9 aerogels are displayed in Figure 1f. Both spectra confirm the presence of the β-phase due to distinctive peaks situated at 840 and 1275 cm^−1^ [41], which are complemented by the characteristic peaks of the γ-phase with maxima at 482 and 1232 cm^−1^ [41]. This analysis revealed that the aerogels prepared with pure DMF as solvent exhibit a mixture of β, and γ phases without evidence of the non-polar α phase at 761 cm^−1^.

*Gelation by addition of a non-solvent to the solution mixture*. It is common to use solvent blends to shift the gelation boundaries of a polymer in solution. The blends can be solvents of different solubility degrees toward the target polymer or solvent/non-solvent mixtures [27], e.g., DMF/ethanol, DMSO/acetone, etc. In the membrane preparation, the solvent mixture has a coagulation agent role [9,42,43]. Based on this principle, aiming to synthesize aerogels at lower PVDF concentrations, a new series of samples were prepared by dissolving 5–12 wt.% of PVDF in a mixture of DMF (solvent, $x_{DMF}=0.5-0.8$) and absolute ethanol (non-solvent, $x_{EtOH}=0.2-0.5$) at 80 °C. Unlike dissolution of PVDF in pure DMF, for a DMF/EtOH blend, wet gels are obtained at concentrations below 9 wt.%. Detailed sample descriptions are displayed in Supplementary Table S1.

To explain this, it is necessary to evaluate the solubility of PVDF in the DMF/ethanol blend. Hansen established an evaluation for a given solvent (or solvent mixture) based on three different intermolecular interactions [44,45]: dispersive or non-polar interactions ($\delta_{D}$), polar interactions between permanent dipoles ($\delta_{P}$), and hydrogen bonds ($\delta_{H}$) [45,46]. Accordingly, Hansen states that a solvent mixture can be represented as a point in a three-dimensional space, the so-called Hansen space, with $\delta_{D}^{m}$, $\delta_{P}^{m}$, and $\delta_{H}^{m}$ coordinates, and can be expressed with the following equation:

(1) $$\delta_{T}^{2}=\delta_{D}^{2,\;m}+\delta_{P}^{2,\;m}+\delta_{H}^{2,\;m}$$

Similarly, a polymer can be represented as a volume in space. Thus, to achieve polymer dissolution, the point that represents the solubility parameter of the solvent blend must be situated within the volume corresponding to the solubility parameter of the polymer [45,47]. To produce such an interaction between the polymer and a solvent blend, Hansen established the relative energy difference (*RED*) parameter that is defined by the polymer/solvent distance ($R_{a}$), and the experimental sphere radii for the polymer ($R_{0}$):

(2) $$RED=\frac{R_{a}}{R_{0}}$$

The parameter of distance is calculated with the following equation [45,46]:

(3) $$Ra={\left(4{\left(\delta_{D,\;P}-\delta_{D,\;S}\right)}^{2}+{\left(\delta_{P,\;P}-\delta_{P,\;S}\right)}^{2}+{\left(\delta_{H,\;P}-\delta_{H,\;S}\right)}^{2}\right)}^{1/2}$$

**Figure 2:**
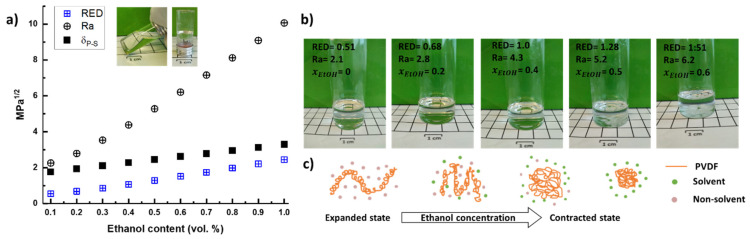
(**a**) RED values at different solvent compositions (blue crossed squares), the distance between the polymer and the solvent coordinates in a three-dimensional Hansen space (crossed circles), and difference of the solubility parameters, as a function of the ethanol content in the solvent mixture (filled squares); (**b**) PVDF dissolved at 80 °C in different solvent blends; (**c**) state of the polymer coils as a function of the ethanol content.

Essentially, the obtained values from these two equations give a simple analogy of solubility in terms of relativeness: for RED < 1, the polymer will dissolve in the blend, and for RED > 1, the affinity between the polymer and the solvent is poor; therefore, the polymer will not dissolve in the blend. In our solvent blend (mixture of DMF and EtOH), the distance ($R_{a}$) between the polymer and the solvent increases progressively with the ethanol (EtOH) content, representing less solubility (Figure 2a). Particularly, the lowest RED and $R_{a}$ values (0.51, and 2.1 MPa^1/2^, respectively) are obtained when PVDF is dissolved in pure DMF, representing the minimum possible difference between the solvent and polymer interaction energies, implying the maximum affinity between polymer and solvent [44]. On the other hand, though the PVDF-solvent affinity gradually decreases as the ethanol content rises, PVDF still solubilizes in the blend in a range of $x_{EtOH}=0-0.4$ with RED < 1. However, as the RED value approaches 1, the boundary condition of PVDF dissolution is reached ($x_{EtOH}=0.4$), and beyond this ethanol content limit ($x_{EtOH}>0.5$), the RED > 1, and partial or non-dissolution occurs, as observed in Figure 2b, displaying non-dissolved PVDF pellets after several hours under stirring and 80 °C.

Upon PVDF dissolution ($x_{EtOH}<0.5$), the polymer chains can experiment different expansion states as a function of the ethanol content; hence, the gelation behavior can vary accordingly (Figure 2c). Namely, at strong PVDF–solvent interactions (i.e., $x_{EtOH}$ ≤ 0.2), the PVDF chains are in an expanded state [48]; then, as the solution is left to stand and cool to room temperature the expanded state is disturbed, and the polymer chains will stack and form nuclei that eventually grow as a spherulite [38]. If the polymer concentration is high enough, the spherulites will build an interconnected backbone; thus, crystallization-induced gelation will take place. This induced gelation effect was observed in all the prepared samples, from 5 to 12 wt.%. In comparison, only the samples with 9 and 12 wt. % PVDF in pure DMF turned into a gel. As $R_{a}$ and RED values steadily increase with the ethanol content, the solubility of PVDF decreases, and the polymer chains tend to contract; hence, the contact between polymer and solvent is minimized. Consequently, the expanded state of the polymer chains shifts to a contractive regime [48,49] favoring nucleation and resulting in crystallization-induced gelation behavior. Experimentally, a low nucleation rate results in slow gelation, whereas fast gelation occurs when a high nucleation regime is reached, as shown by the fast gelation times (Supplementary Table S1).

**Figure 3:**
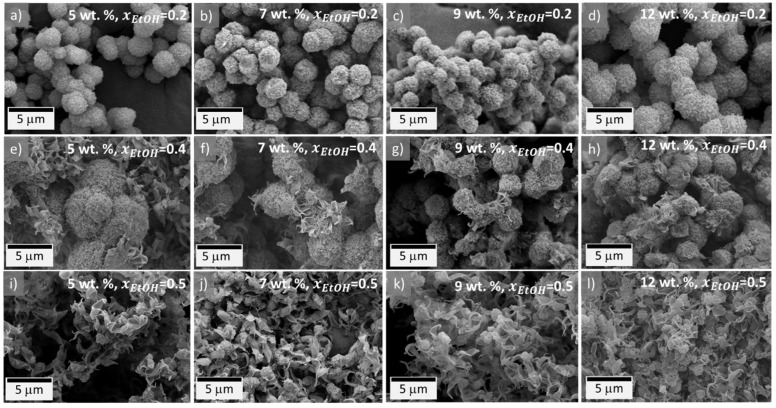
Microstructure of the as-obtained PVDF aerogels, using different DMF/ethanol blends; (**a**–**d**) PVDF aerogels (5–12 wt.%) prepared in a solvent blend containing $x_{EtOH}=0.2$, and $x_{DMF}=0.8$; (**e**–**h**) PVDF aerogels (5–12 wt.%) prepared in a solvent blend containing $x_{EtOH}=0.4$, and $x_{DMF}=0.6$; (**i**–**l**) PVDF aerogels (5–12 wt.%) prepared in a solvent blend containing $x_{EtOH}=0.5$, and $x_{DMF}=0.5$.

*Microstructure and crystallinity***.** The aerogels prepared with low ethanol content ($x_{EtOH}=0.2$, Figure 3a–d) present a similar spherulitic morphology to the aerogels produced in pure DMF. This supports the calculated $R_{a}$ and RED values, showing that, at this composition, the blend behaves as a good solvent for PVDF, and the polymer chains are thus giving enough time for stacking and generating the characteristic spherulitic morphology. Additionally, this ethanol content produces enough nucleation sites to promote crystallization-induced gelation. This equilibrium is no longer effective with an excess of nucleation sites ($x_{EtOH}=0.4$) since, at an initial state, the polymer will form spherulites, but as the amount of dissolved PVDF in the solution decreases, rapid nucleation takes place, and the remaining polymer will grow as leaf-like particles on the surface of the previously formed spherulites, as observed in Figure 3e–h. This effect is more visible at the highest ethanol content, as observed in the micrographs displayed in Figure 3i–l. At these conditions, the contraction of the chains is so strong that they will immediately nucleate and will exclusively form leaf-like particles with some signs of tiny spheres. In summary, DMF allows for a complete dissolution and expansion of the polymer chains, resulting in a controlled formation of spherulitic particles, whereas ethanol has a nucleation agent role that promotes gelation. However, at high concentrations, uncontrolled particle growth occurs.

**Figure 4:**
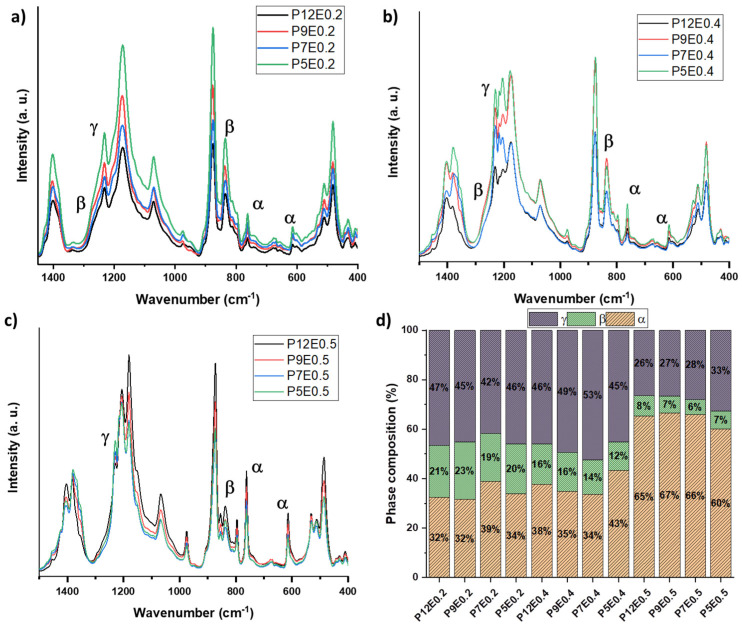
(**a**) FTIR spectra of the PVDF aerogels prepared with a $x_{EtOH}=0.2$; (**b**) FTIR spectra of the PVDF aerogels prepared with a $x_{EtOH}=0.4$; (**c**) FTIR spectra of the PVDF aerogels prepared with a $x_{EtOH}=0.5$; (**d**) quantitative phase composition of all the PVDF aerogels prepared by using DMF as solvent and EtOH as non-solvent.

FTIR analyses were performed to reveal possible compositional variations due to the addition of ethanol (Figure 4a–c). The recorded IR spectra revealed that independently of the PVDF concentration and blend composition, all the samples contain a mixture of the α, β, and γ phases as validated by the most characteristic peaks arising at 761, 840, and 1231 cm^−1^, representative of the α, β, and γ, respectively. Nevertheless, the ethanol concentration seems to influence the relative content of these phases, as observed in Figure 4d. For the samples prepared with $x_{EtOH}=0.2$, the γ-PVDF is the most dominant phase, ranging from 42 to 47%, followed by a considerable amount of α-phase from 32 to 39%, and complemented by a fluctuating 19–23% of β-phase. The same trend was obtained with aerogels prepared with $x_{EtOH}=0.4,$ where the phase dominance is γ>α>β, with slight preference on the formation of γ, and α over β, since the content of the β phase is reduced up to 8%, such as for the samples prepared with 5 wt.%. Important changes were detected for aerogels prepared with the highest ethanol content ($x_{EtOH}=0.5$), as evidenced by the predominance of the α phase with more than a double amount (60–67%) in comparison with the $x_{EtOH}=0.2$ aerogels. The change is accompanied by a proportional reduction of the content of polar phases. This β/γ-to-α phase transition is correlated to the higher contraction of the PVDF chains in the blend promoting the trans-gauche conformation of the thermodynamically more stable α-phase [48]. Furthermore, the polarity of the nucleating agent also plays an important role in the phase composition [50,51]. Based on the large increase in the content of the α phase when PVDF is dissolved in a DMF:EtOH mixture of 1:1, it can be assumed that, under the studied conditions, ethanol promotes the nucleation of α-PVDF crystals.

On the other hand, the enhanced nucleation of the PVDF solutions by the effect of ethanol leads to being able to obtain lower-density and higher-porosity PVDF aerogels ($\rho_{b}$= 0.077 g cm^−3^, and 95%, for the 5 wt.% aerogel; see Supplementary Table S2) in comparison with the samples obtained in pure DMF, where the lowest achieved values were $\rho_{b}$ = 0.093 g cm^−3^ and 93% of porosity for the aerogel with 9 wt.% PVDF (Table 1). However, the P12 and P9 samples have a higher specific surface area, 145 and 168 m^2^ g^−1^, respectively, in comparison with the prepared with ethanol of 113 and 136 m^2^ g^−1^ for P12E0.2 and P9E0.2, respectively; these variations could be related to changes in the particles’ morphologies. In general, the obtained specific surface areas in this work are comparable with the ones reported in the literature of around 100 m^2^ g^−1^ [16,32,52]; nevertheless, some authors reported PVDF–graphene composite aerogels with specific surface areas as high as 200 m^2^ g^−1^, which is attributed to the intrinsic graphene surface area [33].

*Addition of water as a secondary nucleation agent*. Considering ethanol to be a nucleation agent greatly favors the gel transition and the formation of the non-polar α phase, a secondary nucleation compound, such as water, was carefully added to the mixture. To investigate a possible correlation between the water and the phase composition of the PVDF aerogels, a new series of samples was prepared. For this study, 5–12 wt.% of PVDF was dissolved in a fixed solvent blend of DMF with $x_{EtOH}=0.2$ at 80 °C. Upon complete polymer dissolution, different PVDF/H_2_O ratios were added, and the solution was left to stand for gelation; finally, supercritical drying was performed to obtain the aerogels.

**Figure 5:**
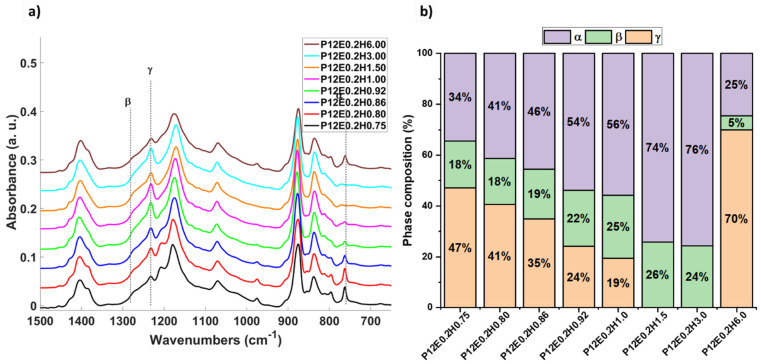
(**a**) FTIR spectra of the aerogels at fixed PVDF concentration of 12 wt.% with varied PVDF/H_2_O ratio from 0.75 to 6; (**b**) crystalline phase composition of the PVDF aerogels.

Figure 5 shows representative FTIR spectra of crystallized PVDF aerogels with water as a secondary nucleation agent at different concentrations. The bands at 614, 761, 795, and 975 cm^−1^, characteristic for the α phase, are observed in the P12E0.2H0.75 (H0.75 corresponds to a PVDF/H_2_0 ratio of 0.75), corresponding to the aerogel with the highest water content. The peaks at 1275 and 840 cm^−1^ indicate the presence of the β phase, and the peak at 1232 cm^−1^ is representative of the γ phase. Figure 5a shows that the content of the α phase is gradually decreasing from 47% proportionally to the water content until no evidence of a phase is present with a PVDF/H_2_O ratio of 1.5. Parallelly, the β-phase and γ-phase associated bands located at 840, and 1232 cm^−1^ replace the α bands, which gain intensity, and become sharper as the water content decreases, indicating a change in the chain conformation. This α-free aerogel is also achieved when a PVDF/H_2_O ratio of 3.0 is used, thus indicating that, under such conditions, it is possible to obtain a monolithic PVDF aerogel with solely β, and γ phases using a blend of $x_{DMF}=0.8$, and $x_{EtOH}=0.2$, with H_2_O as a phase control agent in a PVDF/H_2_O ratio between 1.5 and 3.0.

These results suggest that water hinders the presence of the α phase and therefore has a stabilizing effect on the β and γ phases; however, this is valid until a critical PVDF/H_2_O ratio of 3.0 since, above this ratio, the α-phase abruptly grows (P12E0.2H6.0 with a PVDF/H_2_O ratio of 6.0), since the content of water is not high enough, and the stabilization is diminished, resulting in up to 70% of α phase formation (brown pattern in Figure 5a,b).

Different phenomena can simultaneously occur during the crystallization of the PVDF aerogels when water is added. Firstly, the disparity of the electronegativity values between the fluorine and carbon atoms (4 and 2.5, respectively) generates highly polarized C-F bonds with a strong electric moment of 7 × 10^−30^ Cm [53]. Thus, the strong dipoles of the C-F bonds are easy to align around the C-C backbone by the polar H_2_O molecules, and a more expanded chain coil is twisted. At this stage, triggered by electrostatic attractions, the negatively charged fluorine atoms interact with the positively charged hydrogen atoms of water [50], resulting in a reduction of the critical energy of β, and γ crystal formation [7].

In comparison, water has a slightly larger dipole moment (1.85 D) than ethanol (1.66 D), and considering a very sensitive system, this fine difference provokes a better affinity toward water molecules than the ones of ethanol. These external polar moieties are critical for influencing the formation of the polar crystal phases, as reported when an ionic liquid is used [16]. At such conditions, it can be proposed that the crystallization-induced gelation of PVDF aerogels takes place in three stages: (1) expansion of the polymer chains in the solvent mixture, which leads to (2) the next stage of nucleation where, although the majority of the nuclei formed by the effect of the amount of ethanol is α, there is also a considerable amount of β and γ crystals present; and (3) in the stabilization stage, the water acts as a “stabilizer” of the polar phases, which causes the β and γ phases to be retained.

**Figure 6:**
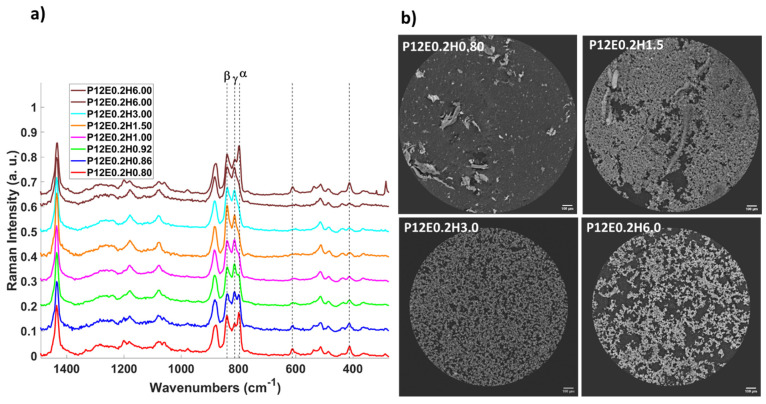
(**a**) Raman spectra of the aerogels prepared with 12 wt.% PVDF, $x_{EtOH}=0.2$, and variable PVDF/H_2_O ratio from 0.80 to 6.0. (**b**) Selected axial maximum intensity projections (MIP) of the aerogels prepared with 12 wt.% PVDF, $x_{EtOH}=0.2$, and variable PVDF/H_2_O ratio from 0.80 to 6.0. The remaining MIP μCT slices are in the SI.

Interestingly, in the sample with the lowest water content (P12E0.2H6.0, PVDF/H_2_O ratio of 6.0), a variable behavior was observed during the Raman analyses since the detected phases varied with the measuring spot, as demonstrated in Figure 6a, where, in a first measurement, the corresponding band of the α-phase is not detected, whereas in a second measurement, the band sharply appears [54,55,56]. This might be a sign of a non-homogeneity attributed to the lack of available water to produce the β- and γ-phase stabilization. Further Synchrotron Radiation Computed micro-Tomography (SR-μCT) measurements were performed on selected samples, as depicted in Figure 6b and Supplementary Figure S1 as axial maximum intensity projections (MIPs). As it can be observed, there is an evident phase-composition change displayed by the predominant presence of dark gray matter observed at a high content of water (from PVDF/H_2_O 0.80 to 1.0, predominant α-phase), whereas practically no dark gray matter can be observed in the samples with a predominance of the β and γ phases (with a PVDF/H_2_O 1.5 to 3.0 ratio), and the small dark voids are porosity-related features, as these are α-free aerogels, as demonstrated by the FTIR and Raman analyses. However, for the MIP μCT slice of the P12E0.2H6.0 aerogel with a predominant α-phase, a considerable dark gray and light gray matter mixture is observed, which we can infer is not only related to the phase composition but is also correlated to the leaf-like particles (dark gray) to the α conformation and the spherulitic microstructure (light gray) to the β/γ conformation.

This is consistent with the morphological features of aerogels containing a considerable amount of α-phase presenting mostly leaf-like particles (e.g., P12E0.4; see Figure 3), and the water-stabilized ones with either α, β, and γ phases (e.g., P12E0.2H0.86 with bimodal leaf-like and spherulitic particles; see Supplementary Figure S2) or pure β/γ aerogels, such as the P12E0.2H1.5 (Supplementary Figure S2), with solely spherulitic morphology.

**Figure 7:**
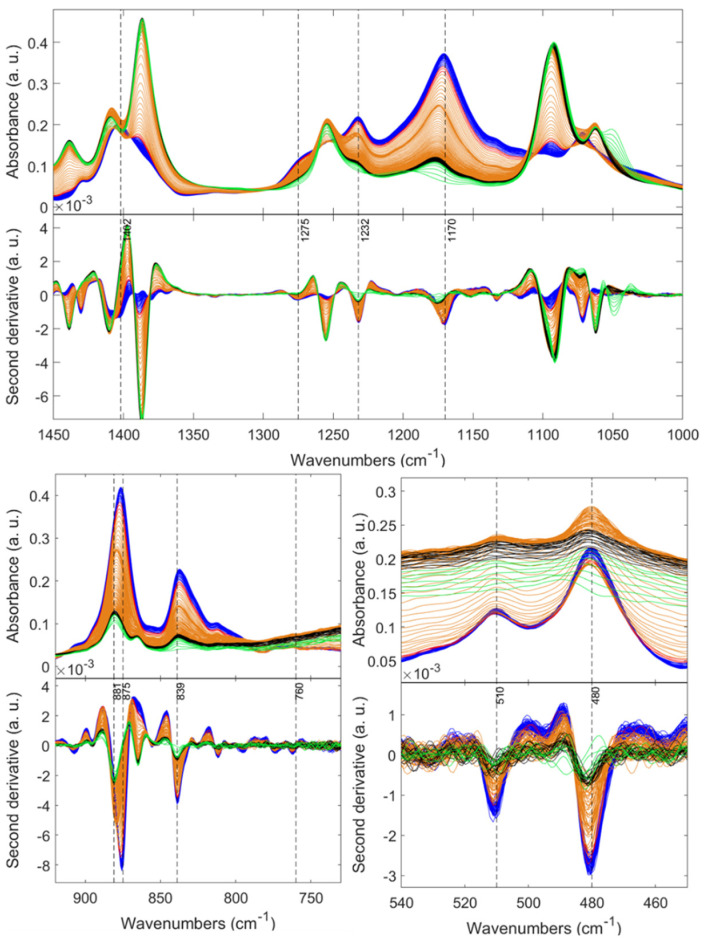
In situ FTIR spectra of the P12E0.2H1.5 solution during the cooling–gelation process (80 °C to RT). For appreciation reasons, the patterns were colored by stages from the beginning to the end: green, black, orange, red, and blue.

*Phase-growth conformation by in situ FTIR*. During the gel transition of the PVDF solutions, the nucleation and growth of crystals take place; hence, significant conformational changes of the PVDF chains may occur during the cooling process. In this regard, in situ FTIR analyses were carried out during the cooling process on two freshly prepared solutions: (1) P12E0.2H1.5 (pure β and γ) and (2) P12E0.2H0.86 (high content α). As displayed in Figure 7, at the beginning of the cooling process (green patterns) of the solution P12E0.2H1.5, most of the detected bands located at 1437, 1406, 1387, 1255, and 1060 cm^−1^ are consistent with the DMF [57]. Exceptionally intense and clearly defined bands emerge at 1402 and 1070 cm^−1^, and these are associated with the wCH_2_-$v_{a}$(C-C) and $v_{a}$(C-C) vibrational modes, respectively [58]. Likewise, the $v_{s}$(C-C) + $v_{s}$(CF_2_) band at 881 cm^−1^ and the duplet bands located at 480 and 510 cm^−1^, correlated to the δ(C-F_2_), and δ(C-F_2_) + w(C-F_2_) [58,59], respectively, are present from the beginning of the cooling process. However, some authors ascribe these bands to any of the phases [41], or they see them as being simply common to the PVDF chain [41]. According to our results, these bands could be exclusively correlated to the polymer and not evidence any crystalline phase, as they are present from the beginning of the nucleation process without evidence of individual chain conformations or possible structural variations recognized by the most unique α, β, or γ bands (761, 1273, and 1232 cm^−1^, respectively). However, broad and poorly defined β and γ bands start to develop with time (green-black patterns). Concretely, the exclusive β band was detected after 50 s at 1275 cm^−1^, with a gradual absorbance intensity increase indicating the progress of the crystalline phase, as it does the strong band at 840 cm^−1^. On the other hand, the γ phase (1232 cm^−1^) is present spontaneously after approximately 50 s, and the absorbance of the band steadily intensifies until its stabilization with no apparent changes after approximately 30 min.

**Figure 8:**
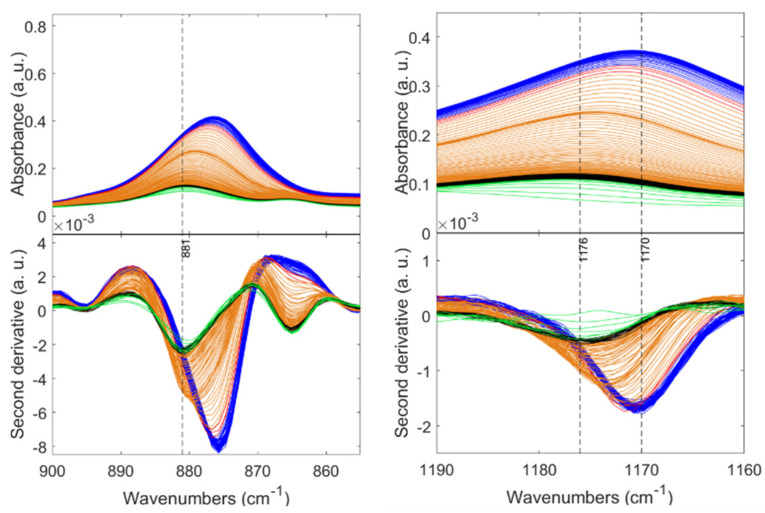
In situ FTIR spectra of the P12E0.2H1.5 solution during the cooling–gelation process (80 °C to RT). For appreciation reasons, the patterns were colored by stages from the beginning to the end: green, black, orange, red, and blue.

Interestingly, as the crystallization of the PVDF proceeds, the initial band at 881 cm^−1^ continues narrowing along with an evident shifting to 876 cm^−1^, as shown in Figure 8. Similarly, this shifting exists in the new band displayed as a broad band at 1177 cm^−1^ within the first 50 s that grows and stabilizes at 1170 cm^−1^ corresponding to the $v_{s}$(CF_2_) + t(CH_2_) vibrational mode. These two bands exhibiting a displacement are characteristic of the polymeric chain and not of a particular phase [58,59,60].

**Figure 9:**
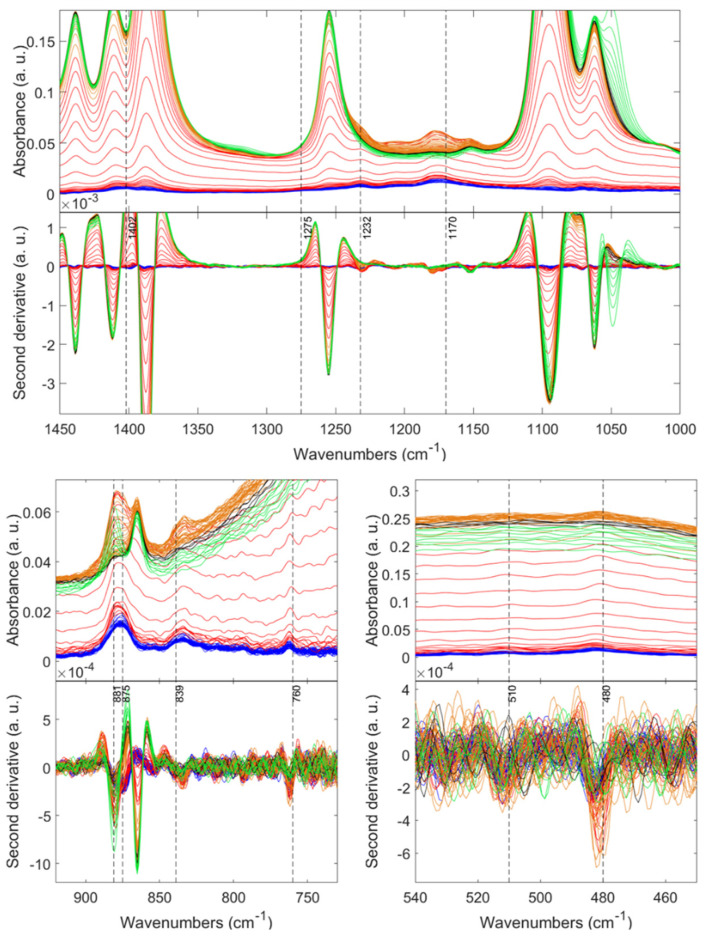
In situ FTIR spectra of the P12E0.2H0.86 solution during the cooling–gelation process (80 °C to RT). For appreciation reasons, the patterns were colored by stages from the beginning to the end: green, black, orange, red, and blue.

Likewise, the typical bands of the α, β, and γ phases appear after a few seconds, accompanied by a constant absorbance intensification proportional to the time. However, they do not present changes concerning their position in the spectrum during the entire crystallization process. Therefore, it is proven that certain segments of the polymer show variations as crystallization advances (such as chain orientation, surface scattering, intermolecular interactions, etc. [61]), and the exclusive segments of the crystalline phases do not rearrange at the given conditions. On the other hand, in the sample with high content of the non-polar α-phase, all the polymer and phase-related peaks appear spontaneously since the beginning of the crystallization process without evidenced preference for a polymer segment. Unlike a peak shifting of the band at 881 cm^−1^, as evidenced when β and γ are present as in the P12E0.2H1.5 sample, the P12E0.2E0.86 displayed a broad band in such a range without shifting, as evidenced in Figure 9.

This is an indication of how the complex nucleation, growth, crystallinity, and eventually the final phase composition can be affected by the sample processing. In other words, such variations in certain polymer chain segments could be attributed to intermolecular changes prompted by changes in chain orientation, molecular interactions, or the PVDF order–disorder transition throughout the time toward gel transition. Particularly, the evidence of this disorder-to-order transition being inherent in the phase composition (P12E0.2H1.5: high polar phases content) comes from the shifting and narrowing of different bands, whereas other reports indicate different transitions as a function of the sample processing [59,62].

## 3. Conclusions

In this work, the feasibility of obtaining PVDF aerogels with adjustable morphology and phase composition was demonstrated. It was found that the ethanol content in the solvent mixture plays a significant role in the development of the microstructure of the aerogel samples since, at low ethanol content, spherulitic particles are obtained, while an increased ethanol content promotes leaf-like particles. Additionally, ethanol as a non-solvent considerably enhances the nucleation and therefore facilitates the gel transition; however, it develops the α phase. Remarkably, the addition of water as a second non-solvent to the mixture greatly improves the customization of aerogels’ phase composition. Following an adequately controlled synthetic protocol, PVDF aerogels with purely polar β and γ crystalline phases can be obtained. According to the obtained findings, in the mixture PVDF/DMF/ethanol/water, ethanol acts as a nucleation agent, whereas water is a phase-stabilizer compound. In this regard, the studied approach is a useful method to synthesize PVDF aerogels with varied physicochemical properties and is believed to be possible to replicate in different PVDF systems.

## 4. Materials and Methods

*Materials.* Polyvinylidene fluoride (PVDF) (MW: ~180,000, MN: 71,000) was purchased from Sigma-Aldrich (Germany). Before use, the PVDF pellets were dried at 80 °C for 24 h. N,N-Dimethylformamide (DMF) AR and absolute ethanol (EtOH) were purchased from VWR (Germany).

*Synthesis of the aerogels*. PVDF gels were prepared by dissolving the polymer in DMF at 80 °C, under vigorous stirring, for 30 min, at concentrations ranging from 5 to 12 wt.%; when required, the PVDF was dissolved in a DMF/EtOH mixture, followed by the addition of H_2_O, and kept stirring for 30 min. Then the solution was poured into sealed plastic containers and left to stand, allowing crystallization-induced gelation for 24 h. The aged gels were submerged in ethanol to remove residual reagents and cycled three times every 24 h. Finally, the gels were dried under supercritical conditions of CO_2_ at 60 °C and 100 bars. Sample labeling is based on the following format: PXEY, where P was selected for the polymer; the subsequent number, X, is the concentration of PVDF in wt.%; E is for ethanol; and the number next to it (Y) represents the ethanol fraction (i.e., P12E0.2 is a sample of 12% wt. PVDF and a $x_{EtOH}=0.2$; the rest is DMF content). When referring to a series of samples with varied content of H_2_O, the polymer/H_2_O ratio is defined as H being the number of the PVDF/H_2_O ratio (i.e., P12E0.2H0.86). The complete sample description is given in Supplementary Table S1.

*Characterization*. The microstructure of the aerogels was studied by using a scanning electron microscope (SEM), Zeiss Ultra Plus. Samples for the SEM studies were placed onto a conductive carbon tape, followed by sputtering of a thin gold layer. The textural properties of the aerogels were determined by nitrogen adsorption–desorption measurements, using a Sy-Lab Micrometrics ASAP 2420 sorption analyzer at 77 K. The samples were degassed at 80 °C for 24 h. MicroActive 5.0 software was used for all calculations based on the obtained isotherms. The specific surface area (S_BET_) was calculated by using the Brunauer–Emmett–Teller (BET) method [63] (using 5 points) in a linear range of 0.05 ≤ *p/p*_0_ ≤ 0.3. Pore size distributions and pore volumes were derived from the desorption branches of the isotherms, using the Barett–Joyner–Halenda (BJH) method.

The bulk density ($\rho_{b}$) of the aerogel monoliths was obtained by using the following equation:

(4) $$\rho_{b}=\frac{m}{v}$$

where m is the mass in grams; and v is the volume, in mL, calculated from the dimensions of the aerogel. The skeletal density ($\rho_{s}$) was obtained by using a helium pycnometer, ULTRAPYC 1200e Quantachrome. The overall porosity (θ) of the selected samples was calculated according to the following equation:

(5) $$\theta =1-\frac{\rho_{b}}{\rho_{s}}$$

Fourier-Transform Infrared Spectroscopy (FTIR) patterns were acquired directly from the as-prepared aerogels. The spectra were taken in a Vertex 70 Bruker spectrometer, from 400 to 1500 cm^−1^. A detailed description of the quantitative analyses is shown in the Supplementary Materials.

*Synchrotron Radiation Computed micro-Tomography.* Synchrotron Radiation Computed micro-Tomography (SR-μCT) acquisitions were performed at SYRMEP beamline, Elettra-Sincrotrone Trieste [64]. Experiments employed the white-beam configuration and the electron storage ring, operating at 2.0 GeV, with a beam current of 308.1 mA. Two filters (1.5 mm Si and 1.0 mm Al) were added to obtain an average energy spectrum of 23.4 keV. To exploit the propagation-based imaging modality, the sample was placed 15 cm from the detector, an Orca Flash 4.0 SCMOS coupled with a 17 μm GGG scintillator. The pixel size was set at 0.9 μm. For each scan, 1800 projections were acquired for 100 ms exposure time per projection during the sample rotation over 180°. SR-μCT slices were reconstructed by using SYRMEP Tomo Project (STP) software [65]. Projections were preprocessed by a phase-retrieval algorithm [66], and then a Filtered Back Projection (FBP) reconstruction algorithm with a ram-lak filter was applied. For every stack, a set of 30 SR-μCT slices were postprocessed by obtaining a 27 μm–thickness Maximum Intensity Projections (MIP) image.

*Raman spectroscopy*. Raman measurements were carried out at the IUVS beamline in Elettra Sincrotrone Trieste [67]. A 266 nm laser was used as the excitation source, and the beam power was 130 μW, with a spot size on the sample around 100 μm. The scattered light was collected through a backscattering configuration. A single-stage 750 mm Czery-Turner spectrometer (Andor SR-750-A) equipped with 1800 grooves/mm holographic grating and a Peltier-cooled CCD (Andor DU420A-BU2) was used to obtain the Raman spectra. The final spectra were obtained by averaging 24 spectra, each with a 5 min integration time, for a total acquisition time of 2 h per sample. A small subsample of each type of the aerogels (P12E0.2H0.8- P12E0.2H6.0) was placed under the beam to be measured.

*In situ Attenuated Total Reflectance Fourier-Transform InfraRed (ATR–FTIR) spectroscopy.* Experiments were carried out at the SISSI-Bio beamline at Elettra-Sincrotrone Trieste [68]. Spectra were acquired by using a Bruker Vertex70v interferometer, coupled to a Wide range FIR MIR beamsplitter and DTGS detector with a Bruker Platinum monolithic diamond ATR setup. A spectrum was acquired every 25 s, with 16 scans, at a spectral resolution of 2 cm^−1^, with a spectral range of 6000–300 cm^−1^. Samples P12E0.2H1.5 and P12E0.2H0.86 were prepared next to the instrument at a constant temperature of 80 °C, a drop of 3 μL was placed onto the ATR crystal, and the measurement started. Crystallization started when it was placed in the ATR crystal because of the decrease in temperature. Spectra were acquired continuously for more than 30 min until they were not showing any changes. The ATR crystal was kept at a constant temperature of 28 °C.

*ATR–FTIR spectroscopy of aerogels:* Experiments were carried out at the SISSI-Bio beamline at Elettra-Sincrotrone Trieste, and spectra were acquired by using a Bruker Vertex 70 interferometer coupled to a DTGS detector, with a single reflection diamond ATR setup GLADI-ATR (Pike Inc). Spectra with 128 scans were recorded with a spectral range of 5000–600 cm^−1^, with a spectral resolution of 4 cm^−1^. Spectra were area normalized over the whole range. A small piece of the aerogel was placed onto the ATR plate and pressed to have better contact.

## Figures and Tables

**Table 1:** Textural properties of the PVDF aerogels.

Sample	S_BET_ (m^2^ g^−1^) ^a^	Pore Diameter (nm) ^b^	Pore Volume (cm^3^ g^−1^) ^c^	t_gel_ min ^d^	$\rho_{b}$ (g cm^−3^) ^e^	$\rho_{s}$ (g cm^−3^) ^f^	Porosity (%)
P12	145	23	0.80	30	0.123	1.47	92
P9	168	23	0.99	45	0.093	1.39	93
P7	-	-	-	No gel	-	-	-
P5	-	-	-	No gel	-	-	-
P3	-	-	-	No gel	-	-	-

^a^ Specific surface area obtained by N_2_ sorption, using the BET method. ^b^ Determined from the desorption curve, using the BJH method. ^c^ Determined from the adsorption curve, using the BJH method. ^d^ Gelation time. ^e^ Bulk density calculated by dimensions and weight. ^f^ Skeletal density calculated by using a He pycnometer.

## Supplementary Materials

The following supporting information can be downloaded at https://www.mdpi.com/article/10.3390/gels8110727/s1. Figure S1: Axial maximum intensity projections. Figure S2: SEM micrographs of the PVDF aerogels containing different particles morphologies. Table S1: Details of the prepared samples. Table S2: Textural properties of the PVDF aerogels prepared by using ethanol as nucleating agent [22,69].
