# Supplementary material for: Polyvinylidene Fluoride Aerogels with Tailorable Crystalline Phase Composition
Source: Gels. 2022 Nov 9;8(11):727. doi: 10.3390/gels8110727 (PMC9689208; doi:10.3390/gels8110727)
Supplement: Supplementary file 1 [file gels-08-00727-s001.zip › gels-2012097-supplementary.pdf]

Article

Polyvinylidene Fluoride Aerogels with Tailorable Crystalline Phase Composition

Jorge Torres Rodriguez [1,2], Diana E. Bedolla [3,4], F. D´Amico[3], Ann-Kathrin Koopmann[1,2], Lisa Vaccari [3,4], Giulia Saccomano[3,5], Richard Kohns[1,2], and Nicola Huesing [1,2,*]

[1] Paris-Lodron University of Salzburg, Department of Chemistry and Physics of Materials, Jakob Haringer-Str. 2A, 5020 Salzburg, Austria
[2] Salzburg Center for Smart Materials, Jakob Haringer-Str. 2A, 5020 Salzburg, Austria
[3] Elettra-Sincrotrone Trieste, SS14 Km 163.5, 34149 Trieste, Italy
[4] Area Science Park, Padriciano 99, 34149 Trieste Italy
[5] Department of Engineering and Architecture, University of Trieste, Via Alfonso Valerio 6/1, 34127 Trieste, Italy
* Correspondence: nicola.huesing@plus.ac.at

Table S1. Detailed description of the prepared samples and their compositions

Sample	PVDF (wt.%.)	PVDF/H_2O	x_{DMF}	x_{EtOH}	t_{gel} (min)
P12	12				30
P9	9				45
P7	7	0	1	0	-
P5	5				-
P3	3				-
P12E0.2	12				21
P9E0.2	9	0	0.8	0.2	23
P7E0.2	7				27
P5E0.2	5				45
P12E0.4	12				15
P9E0.4	9	0	0.6	0.4	18
P7E0.4	7				25
P5E0.4	5				45
P12E0.5	12				10
P9E0.5	9	0	0.5	0.5	12
P7E0.5	7				15
P5E0.5	5				16
P12E0.2H0.75		0.75			3
P12E0.2H0.80		0.80			5
P12E0.2H0.86		0.86			8
P12E0.2H0.92	12	0.92	0.8	0.2	10
P12E0.2H1.0		1.0			10
P12E0.2H1.5		1.5			11
P12E0.2H3.0		3.0			13
P12E0.2H6.0		6.0			18

Table S2. Textural properties of the PVDF aerogels prepared using ethanol as nucleation agent

Sample	S_{BET} (m^2 g^{-1})[a]	Pore diameter (nm)[b]	Pore volume (cm^3 g^{-1})[c]	ρ_b (g cm^{-3})[d]	ρ_s (g cm^{-3})[e]	Porosity (%)
P12E0.2	113	19	0.54	0.125	1.49	92
P9E0.2	136	23	0.80	0.096	1.37	93
P7E0.2	160	21	0.86	0.086	1.58	94
P5E0.2	152	22	0.85	0.077	1.40	95

[a]Specific surface area obtained by N_2 sorption using the BET method
[b]Determined from the desorption curve using the BJH method
[c]Determined from the adsorption curve using the BJH method
[d]bulk density calculated by dimensions and weight
[e]skeletal density calculated using a He pycnometer

The relative content of the crystalline phases $(F(\alpha+\beta+\gamma))$ in the PVDF aerogels was calculated using the following equation [1]:

$$F(\beta+\gamma) = \frac{A_{\beta,\gamma}}{\left(\frac{k_{\beta,\gamma}}{k_\alpha}\right)A_\alpha + A_{\beta+\gamma}} * 100\%$$

Where A_α and A_β are the corrected baseline absorbance at 761 cm^{-1} and 840 cm^{-1}, respectively. k_α is $6.1x10^4 cm^2\ mol^{-1}$ and $k_{\beta,\alpha}$ is $7.7x10^4 cm^2\ mol^{-1}$ are the corresponding absorption coefficients. $F(\beta)$ and $F(\gamma)$ are calculated by using the intensity of the absorption peaks of 1275 cm^{-1} and 1234 cm^{-1}[2], for the β, and γ phases, respectively, according to the following equations:

$$F(\beta) = F(\beta+\gamma) * \frac{A_{1275}}{A_{1275}+A_{1234}} x * 100\%$$

$$F(\gamma) = F(\beta+\gamma) * \frac{A_{1234}}{A_{1275}+A_{1234}} * 100\%$$

Finally, the relative content of α-PVDF $(F(a))$ was calculated using $F(a) = 1 - F(\beta) - F(\gamma)$.

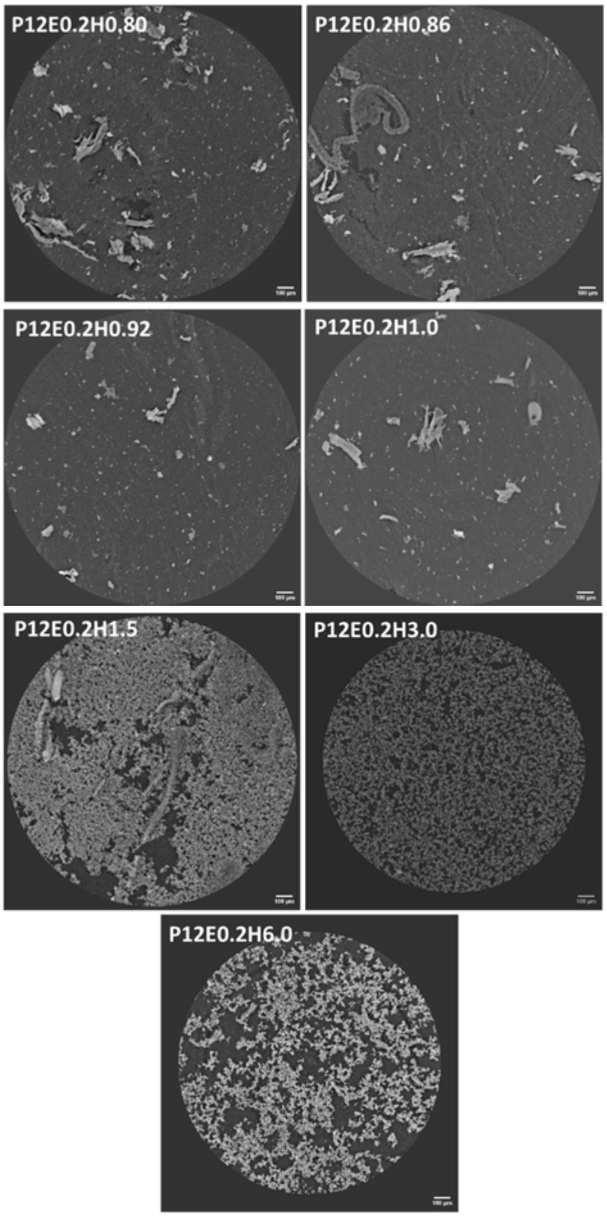

Figure S1. Axial maximum intensity projections (MIP) of the aerogels prepared with 12 wt.% PVDF, $x_{EtOH} = 0.2$, and variable PVDF/H_2O ratio from 0.80 to 6.0.

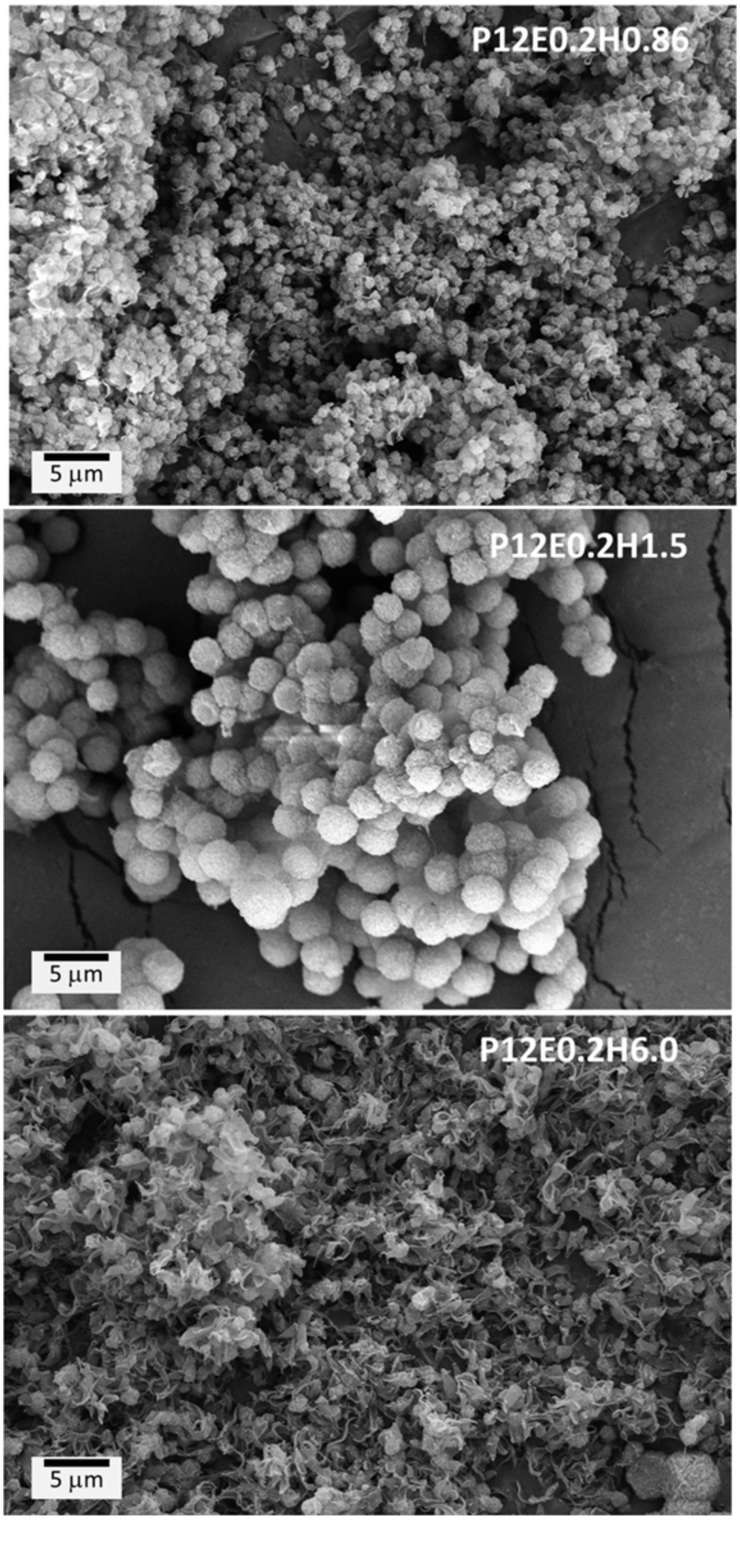

Figure S2. SEM micrographs displaying the morphological differences between the samples with variable phase composition. The P12E0.2 sample has a bimodal morphology of spherulites and leaf-like particles; the P12E0.2H1.5 aerogel has solely spherulites; the P12E0.2H6.0 has mostly leaf-like particles with small signs of tiny spherulites.

1. Zhou, H.; Wang, H.; Liu, Z.; Yang, H.; Yuan, C.; Wang, Y. Facilitated Phase Transformation of PVDF in Its Composite with an Ionic Liquid. *Polymer (Guildf).* **2021**, *220*, doi:10.1016/j.polymer.2021.123564.
2. Gregorio, Jr., R.; Cestari, M. Effect of Crystallization Temperature on the Crystalline Phase Content and Morphology of Poly(Vinylidene Fluoride). *J. Polym. Sci. Part B Polym. Phys.* **1994**, *32*, 859–870, doi:10.1002/polb.1994.090320509.
